# Supplementary material for: Iterative improvement in the automatic modular design of robot swarms
Source: PeerJ Comput Sci. 2020 Dec 7;6:e322. doi: 10.7717/peerj-cs.322 (PMC7924708; doi:10.7717/peerj-cs.322)
Supplement: Supplemental Information 3 [file peerj-cs-06-322-s003.zip › argos3/doc/api/standalone/a00582_source.html]

ARGoS: plugins/simulator/entities/perspective\_camera\_equipped\_entity.cpp Source File


- Main Page
- Related Pages
- Namespaces
- Classes
- Files

- File List
- File Members

# plugins/simulator/entities/perspective\_camera\_equipped\_entity.cpp

Go to the documentation of this file.

```
00001 #include "perspective_camera_equipped_entity.h"
00002 #include <argos3/core/simulator/space/space.h>
00003 #include <argos3/core/simulator/entity/composable_entity.h>
00004 
00005 namespace argos {
00006 
00007    /****************************************/
00008    /****************************************/
00009 
00010    CPerspectiveCameraEquippedEntity::CPerspectiveCameraEquippedEntity(CComposableEntity* pc_parent) :
00011       CEntity(pc_parent),
00012       m_fFocalLength(0.0f),
00013       m_fRange(0.0f),
00014       m_nImagePxWidth(0),
00015       m_nImagePxHeight(0),
00016       m_psAnchor(NULL) {
00017       Disable();
00018    }
00019 
00020    /****************************************/
00021    /****************************************/
00022 
00023    CPerspectiveCameraEquippedEntity::CPerspectiveCameraEquippedEntity(CComposableEntity* pc_parent,
00024                                                                       const std::string& str_id,
00025                                                                       const CRadians& c_aperture,
00026                                                                       Real f_focal_length,
00027                                                                       Real f_range,
00028                                                                       SInt32 n_width,
00029                                                                       SInt32 n_height,
00030                                                                       SAnchor& s_anchor) :
00031       CEntity(pc_parent, str_id),
00032       m_cAperture(c_aperture),
00033       m_fFocalLength(f_focal_length),
00034       m_fRange(f_range),
00035       m_psAnchor(&s_anchor) {
00036       SetImagePxSize(n_width, n_height);
00037       Disable();
00038    }
00039 
00040    /****************************************/
00041    /****************************************/
00042 
00043    void CPerspectiveCameraEquippedEntity::Init(TConfigurationNode& t_tree) {
00044       try {
00045          CEntity::Init(t_tree);
00046          CDegrees cApertureDeg;
00047          GetNodeAttribute(t_tree, "aperture", cApertureDeg);
00048          m_cAperture = ToRadians(cApertureDeg);
00049          GetNodeAttribute(t_tree, "focal_length", m_fFocalLength);
00050          GetNodeAttribute(t_tree, "range", m_fRange);
00051          GetNodeAttribute(t_tree, "width", m_nImagePxWidth);
00052          GetNodeAttribute(t_tree, "height", m_nImagePxHeight);
00053          SetImagePxSize(m_nImagePxWidth, m_nImagePxHeight);
00054          std::string strAnchorId;
00055          GetNodeAttribute(t_tree, "anchor", strAnchorId);
00056          /*
00057           * NOTE: here we get a reference to the embodied entity
00058           * This line works under the assumption that:
00059           * 1. the PerspectiveCameraEquippedEntity has a parent;
00060           * 2. the parent has a child whose id is "body"
00061           * 3. the "body" is an embodied entity
00062           * If any of the above is false, this line will bomb out.
00063           */
00064          CEmbodiedEntity& cBody = GetParent().GetComponent<CEmbodiedEntity>("body");
00065          m_psAnchor = &cBody.GetAnchor(strAnchorId);
00066       }
00067       catch(CARGoSException& ex) {
00068          THROW_ARGOSEXCEPTION_NESTED("Error initializing the perspective camera equipped entity \"" << GetId() << "\"", ex);
00069       }
00070    }
00071 
00072    /****************************************/
00073    /****************************************/
00074 
00075    void CPerspectiveCameraEquippedEntity::Enable() {
00076       if (m_psAnchor) {
00077          m_psAnchor->Enable();
00078       }
00079    }
00080 
00081    /****************************************/
00082    /****************************************/
00083 
00084    void CPerspectiveCameraEquippedEntity::Disable() {
00085       if (m_psAnchor) {
00086          m_psAnchor->Disable();
00087       }
00088    }
00089 
00090    /****************************************/
00091    /****************************************/
00092 
00093    REGISTER_STANDARD_SPACE_OPERATIONS_ON_ENTITY(CPerspectiveCameraEquippedEntity);
00094 
00095    /****************************************/
00096    /****************************************/
00097 
00098 }
```

---

Generated on 10 Jul 2018 for ARGoS by 
 1.6.1
